# Stability indicating RP-HPLC method for estimation of finerenone and its related substances in new dosage form

**DOI:** 10.1038/s41598-025-07166-4

**Published:** 2025-06-20

**Authors:** Aya A. Marie, Mohamed G. Yassin, Eman A. Elshenawy

**Affiliations:** 1 Pharmaceutical Chemistry Department, Faculty of Pharmacy, Horus University, New Damietta, 34517 Egypt; 2 Zeta Pharma for Pharmaceutical Industries, Sadat City, Egypt; 3 Pharmaceutical Analytical Chemistry Department, Faculty of Pharmacy, Tanta University, Tanta, Egypt

**Keywords:** Finerenone, RP-HPLC, Stability indicating, Tablet dosage forms, Related substances, Unspecified impurities, Diseases, Medical research, Chemistry, Mathematics and computing

## Abstract

Stability indicating RP-HPLC method was developed and validated for the estimation of finerenone (FIN) in pure and in new tablet dosage form. The proposed approach was applied and validated for determination of (FIN) related substances. Stability studies of (FIN) was carried out using five stress conditions; acid, alkali, oxidation, photodegradation and heat degradation. The chromatographic analysis of (FIN) was based on using mobile phase consist of filtered and degassed mixture of 450mL water, 550mL acetonitrile and 10mL triethylamine with pH adjusted to 7. Phenomenex (C18, 4.6 × 250 mm, 5 μm) column was used with 0.8 mL/min flow rate and 40 °C column temperature. The UV detection was set at 252 nm with injection volume (10µL). The (FIN) retention time was 4.437 ± 0.05 min. The proposed technique was validated according to ICH guidelines with good linearity in ranges (8–30 µg/mL) for assay of (FIN) and (0.2–1.4 µg/mL) for determination of (FIN) unspecified impurities. The found mean percentage recoveries were 99.74% for (FIN) assay and 99.11% for (FIN) related substance determination which indicate good trueness. The developed approach was successfully applied for the determination of (FIN) in Nexifinerenone^®^ film coated tablet dosage form. Good agreement was established when assay results using the validated RP-HPLC method were compared statistically to those obtained using the reported method. For the greenness assessment Complex GAPI, Complex MoGAPI and AGREE methods were applied.

**Supplementary Information:**

The online version contains supplementary material available at 10.1038/s41598-025-07166-4.

## Introduction

Chronic kidney disease (CKD) is considered as a worldwide health problem^1^. The efficacy of finerenone (FIN) for chronic kidney disease treatment has been the issue of considerable debate in recent years^2^. (FIN), (**Figure **S1) is a first-in-class, orally administered non-steroidal mineralocorticoid receptor blocker^3^. It has been used for treating the chronic heart failure with reduced ejection fraction^4^. It has been approved in the USA to decrease the risk of cardiovascular death, end stage renal illness, heart failure hospitalization in patients of (CKD) with diabetes type 2 ^5^.

Nexifinerenone^®^ film coated tablet (FCT) for treatment of heart failure and diabetic kidney disease. Nexifinerenone^®^ present in two strengths 10 mg (FIN) and 20 mg (FIN) per tablet. Nexifinerenone^®^ film coated tablet (FCT) was manufactured by Zeta Pharma for Pharmaceutical Industries.

Only few methods have been reported for analysis of (FIN) including UV-spectrophotometry^6^LC-MS^7,8^RP-HPLC^8–14^ and one fluorescence chemo sensor approach^15^. By reviewing all published RP-HPLC methods, it has been proven that there are stability indicating methods reported for estimation of (FIN)^8,10,12,13^. However, no stability indicating approaches have been published for determination of unspecified impurities in (FIN) tablets. The proposed RP-HPLC approach was established for determination of (FIN) without needing sophisticated and tedious procedures or instrumentation like LC–MS^7^. Recent studies have highlighted significant advancements in sampling and sorbent-based sample preparation techniques, particularly in bioanalytical contexts^16^. Related substances are impurities deriving from the drug substance. Related substances including manufacturing process impurities, synthetic impurities and degradation products^17^. Related substances in drug product are associated to pharmaceutical efficacy, stability and safety. An impurity defined by the (ICH) guidelines as the component of the drug substance which is not the chemical entity defined as the drug substance^18^. Only impurities that are probably to be existing in considerable level in the final drug product should be considered^17,19^. Impurities are known and unknown based on knowledge of its chemical composition. The drug safety is based not only on the toxicological characters of the active ingredient, but also on the impurities which it containing. So, the identification and determination of impurities in the pharmaceutical product, are very important^18^. For any “unknown” impurity (not belonging to the potential impurities), reporting and identification thresholds are considered important to be applied according to ICH Q3(B) guidelines^17^. In the final pharmaceutical product, the impurities that are detected in level exceed the reporting thresholds are the only impurities that can be considered as actual impurities according to ICHQ3(B) guidelines^17^. The identification is essential only when the levels of these impurities are expected to be above the specification or identification thresholds in the final drug product^17^. The Determinations of unidentified impurities is very important as the total amount of unidentified impurities is usually compared to or sometimes more than the total amount of identified impurities.

The aim of this work is to establish and validate a stability indicating RP-HPLC approach for quantitation of (FIN) in bulk and new tablet dosage form in addition, applying this approach for the determination of unspecified impurities in drugtablets Nexifinerenone^®^ 10 mg/tablet. Good agreement was established when the assay results of (FIN) using the established RP-HPLC method were compared statistically to the assay results obtained using the published one^9^. Complex GAPI^20^Complex MoGAPI^21^ and AGREE^22^ methods were applied for assessment of the greenness of the proposed method^23^.

The novelty of this study lies in the development and validation of the first stability-indicating RP-HPLC method specifically for the detection and quantification of FIN related substances in a newly developed tablet dosage form. Unlike previously published methods, which were limited to FIN assay or required complex instrumentation like LC-MS, this method provides a simple, cost-effective, and robust alternative that complies with ICH Q3B guidelines for impurities. It successfully differentiates FIN from its degradation products under various stress conditions, ensuring accurate detection of the unknown impurities, which is crucial for ensuring drug safety, efficacy, and regulatory compliance.

## Materials and methods

### Apparatus and software

Shimadzu prominence-i^®^ series LC-2030 C 3D plus system (Shimadzu, Kyoto, Japan) was used with RS auto-sampler injector, quaternary RS pump and thermostated RS column compartment. Lab solutions DB version 6.111 software^®^ (Shimadzu, Japan) was used for data acquisition. Phenomenex ODS (250 mm, 4.6 mm, 5µ) column. Jenway 3510 pH-meter (UK) and Hettich centrifuge (Tuttlingen, Germany). Rocker 811 lab vacuum pump (Lingya Dist., Kaohsiung, Taiwan) and nylon membrane filter 0.22 μm (Millipore, Ireland) were used.

Different software was used for the greenness assessment; the software for the Complex GAPI is available under the open-source MIT license and can be downloaded from https://mostwiedzy.pl/complexgapi. For the AGREE method freely available Beta version of software was used for the procedures assessment. It is open-source and downloadable from https://mostwiedzy.pl/AGREE. The software used for the MoGAPI tool is also freely available (open source) at https://fotouhmansour.github.io/MoGAPI/ that facilitate application and method comparison.

### Chromatographic conditions

Chromatographic separation was performed using Phenomenex ODS (250 mm, 4.6 mm, 5µ) column. The mobile phase was filtered and degassed mixture of 450mL water, 550mL acetonitrile and 10mL triethylamine with pH adjusted to 7. UV detection was at 252 nm, 0.8mL/min flow rate with (10µL) injection volume and 40 °C column temperature.

### Materials and reagents

(FIN) (purity 99.60%) and Nexifinerenone^®^ (FCT) were obtained friendly from Zeta Pharma for Pharmaceutical Industries (El Menofia, Egypt). Nexifinerenone^®^ tablets available in Egyptian markets in two strengths (10 mg and 20 mg) (FIN) per tablet.

HPLC grade acetonitrile and ethanol (Fisher, UK), analytical grade triethylamine and DMSO (Sigma-Aldrich), HCl (Oxford Laboratory Reagent), NaOH (ISO-CHEM laboratory reagent) and H_2_O_2_ (Lab Chem) were used.

### Stock and working standard solutions

For preparation of stock standard solution 200 µg/mL (FIN), accurate weight 20 mg of (FIN) was transferred into 100mL volumetric flask. 5mL DMSO were used to dissolve the drug with sonication for (1 min). Then 50mL ethanol (96%) was added and sonicate for 5 min with intermediate shake and then ethanol was added up to volume.

For preparation of working solution 5mL of the stock solution was diluted into 10mL volumetric flask with ethanol and completed to the mark to attain 100 µg/mL (FIN). All solutions were stable for one month when kept at 4 °C.

### Construction of calibration curves

Different aliquots were transferred from 100 µg/mL (FIN) working solution into separate 10mL volumetric flasks. The solutions were diluted with mobile phase to obtain concentration ranges (8–30 µg/mL) for the assay and (0.2–1.4 µg/mL) for testing of unspecified impurities. 10µL were injected from each solution using the specified chromatographic separation conditions. Construction of calibration curves by plotting the average peak area against (FIN) concentrations and the regression equations were computed.

### Procedure for Preparation of nexifinerenone^®^ tablets

In a mortar, ten Nexifinerenone^®^ tablets 10 mg/tablet were weighed and ground into a fine powder. Then into 100mL volumetric flask a weight of powdered tablet equivalent to the average weight of one tablet was transferred, 5mL water were added with sonication and shaking to disperse the powder. Then 5mL DMSO were added and mix well. 50mL ethanol were added with sonication and shaking for (10 min). The solution was cooled then diluted up to the mark with same solvent. At (5000 rpm) the solution was centrifuged for 10 min. Then into 25mL volumetric flask 5mL supernatant was transferred and diluted to the mark to attain 20 µg/mL (FIN) using mobile phase.

### Solutions for testing the presence of unspecified impurities

For determination of unspecified impurities three solutions were prepared. The concentration of reporting and identification threshold solutions depend on the maximum daily dose of (FIN) which is 20 mg/mL. The reporting threshold was (0.1% of the test solution concentration) and identification threshold was (0.2% of the test solution concentration) according to the maximum daily dose of (FIN). A solution of 0.5 mg/mL (FIN) was prepared as test solution, 0.0005 mg/mL (FIN) reporting threshold solution (0.1%) and 0.001 mg/mL (FIN) as identification (specification) threshold solution (0.2%).

#### Solution 1:( test sample solution)

In 100mL volumetric flask a weight of powdered tablet equivalent to 50 mg of (FIN) was transferred and 5mL of water was added with sonication and shaking till tablets disintegrated. Then 5mL DMSO were added with sonication for (5 min). 50 mL ethanol (96%) was added with sonication again for (10 min) with intermediate shake. The resulted solution was mixed well and diluted up to volume with ethanol to obtain (0.5 mg/mL). At (5000 rpm) the solution was centrifuged for (5 min) and (10µL) of supernatant was injected to the chromatograph.

#### **Solution 2: (Identification or specification limit) (0.2%)**

Identification or specification threshold solution was prepared from stock solution 200 µg/mL (FIN). This solution was prepared by transferring 5mL of stock solution into 100mL volumetric flask, then diluted and completed to volume with ethanol. From the resulted solution, 5mL were transferred into 50mL volumetric flask. Ethanol was used to complete the volume to attain (0.001 mg/mL).

#### Solution 3: (Reporting Threshold) (0.1%)

Into 10mL volumetric flask, 5mL of (solution 2) was transferred and diluted up to the mark using ethanol to obtain (0.0005 mg/mL).

## Forced degradation studies solutions

The specificity of the developed approach was recognized based on stability studies which were confirmed by comparing the unstressed 20 µg/mL (FIN) chromatogram for assay and 0.5 mg/mL (FIN) for impurities determination with degradation solutions.

### Stability studies for assay

The preparation of non-stressed (FIN) solution for assay was carried out by transferring 5mL of working standard solution 100 µg/mL (FIN) into 25mL volumetric flask and diluted to volume with ethanol to obtain 20 µg/mL (FIN).

The comparison was performed between the non-stressed (FIN) solution and degradation solutions by the presence of new degradation peaks and/or the decrease in the peak area of (FIN).

#### Alkaline hydrolysis

Into 25mL volumetric flask containing 5mL 0.1 N NaOH, 5mL of 100 µg/mL (FIN) working solution were transferred and stand for 2 h. at 80 °C for alkaline degradation investigation.

After the mentioned time the solution was neutralized by adding 5mL of 0.1 N HCl and diluted to the mark using ethanol to obtain 20 µg/mL (FIN).

#### Acid degradation

Into 25mL volumetric flask containing 5mL of 0.1 N HCl, 5mL from 100 µg/mL (FIN) working solution were transferred and stand for 2 h. at 80 °C for investigation of acidic degradation.

After specified time the solution was neutralized by adding 5mL of 0.1 N NaOH and diluted to the mark using ethanol to obtain 20 µg/mL (FIN).

#### Oxidation

From 100 µg/mL (FIN) working solution, 5mL was transferred in 25mL volumetric flask contained 0.5mL of 30% H_2_O_2_. This solution was stand for 30 min at room temperature for investigation of oxidation degradation. After specified time, the solution was diluted to the mark using ethanol to attain 20 µg/mL (FIN).

#### Photo degradation

5mL of 100 µg/mL (FIN) were transferred into 25mL volumetric flask for photodegradation examination. The solution was diluted to the mark using ethanol to attain 20 µg/mL (FIN). The resulted solution was exposed to the day light for 24 h. After the specified time 10µL was injected under specified separation conditions.

#### Heat degradation

5mL of 100 µg/ml (FIN) was transferred into 25mL volumetric flask. Then solution was diluted up to the mark using ethanol. For investigation of heat degradation this solution was exposed to heat at 80 °C for 2 h.

### Stability studies for unspecified impurities determination

Comparison was performed between the non-stressed 0.5 mg/mL (FIN) solution of with degradation solutions by measuring the decrease in the (FIN) peak area and/ or the presence of new degradation peaks.

#### Acid degradation

A weight of powdered tablets equivalent to 50 mg of (FIN) was transferred into 100mL volumetric flask for investigation of acidic degradation. Then 5mL of water was added with sonication and shaking till tablets disintegrated. Then 5mL DMSO was added with sonication for 5 min. Then 5mL of 0.1 N HCl was added for 2 h. at 80 °C. After mentioned time this solution was neutralized by adding 5mL of 0.1 N NaOH and diluted up to the mark using ethanol to obtain 0.5 mg/mL (FIN).

#### Alkaline hydrolysis

A weight of powdered tablets equivalent to 50 mg of (FIN) was transferred into 100mL volumetric flask for investigation of alkaline degradation. Then 5mL of water was added with sonication and shaking till tablets disintegrated. Then 5mL DMSO was added with sonication for 5 min. Then 5mL of 0.1 N NaOH was added for 2 h. at 80 °C. After mentioned time this solution was neutralized by adding 5mL of 0.1 N HCl and ethanol was used to complete the solution up to the mark to obtain 0.5 mg/mL (FIN).

#### Oxidation

A weight of powdered tablets equivalent to 50 mg of (FIN) was transferred into 100mL volumetric flask for investigation if oxidation degradation. Then 5mL of water was added with sonication and shaking till tablets disintegrated. Then 5mL DMSO was added with sonication for 5 min. The resulted solution was exposed to 0.5mL of 30% H_2_O_2_ for 30 min at room temperature. The ethanol was used to complete the solution up to the mark to attain 0.5 mg/mL (FIN).

#### Photo degradation

For investigation of photodegradation, a weight of powdered tablets equivalent to 50 mg of (FIN) into volumetric flask 100mL. Then 5mL of water was added with sonication and shaking till tablets disintegrated. Then 5mL DMSO was added with sonication for 5 min. The solution was diluted up to mark with ethanol to obtain (0.5 mg/mL). The resulted solution was exposed to the day light for 24 h. After the specified time, (10µL) was injected under the specified chromatographic conditions.

#### Heat degradation

A weight of powdered tablets equivalent to 50 mg of (FIN) was transferred into 100mL volumetric flask for investigation of heat degradation. Then 5mL of water was added with sonication and shaking till tablets disintegrated. Then 5mL DMSO was added with sonication for 5 min. The solution was diluted up to the mark using ethanol to obtain (0.5 mg/mL) then exposed to heat at 80 °C for 2 h.

## Results and discussion

**Fig. 1:**
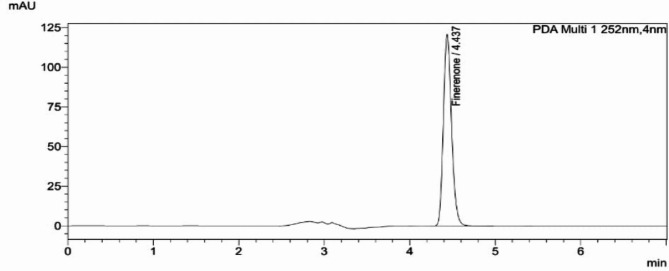
Typical chromatogram of 20 µg/mL (FIN) under the specified chromatographic conditions.

The developed SIAM method was applied successfully for estimation of (FIN) in their bulk and new tablet dosage forms. The retention times of (FIN) was 4.437 ± 0.05 min as shown in Fig. 1.

### Method development and optimization

Numerous parameters such as pH of mobile phase, mobile phase flow rate, volume of organic modifier and column temperature were known to affect the number of theoretical plates, capacity factor, retention time and peak shape. So, the method optimization was performed based on varying all these conditions with evaluation of their effects on different parameters to choose the best chromatographic conditions. The mobile phase pH was varied in range (3–7) at constant column temperature 40 °C, acetonitrile volume 550mL and 0.8mL/min flow rate. The mobile phase pH 7 was chosen for optimum retention time and peak shape. Log p of (FIN) is 2.2, this indicating that it is a lipophilic molecule. As the pk_a_ of (FIN) is 6.06 (strongest basic) so C_18_ column was selected with pH 7 to suppress the ionization of (FIN) and increase its retention on the column.Also, the dimensions of column are intended to increase the number of theoretical plates. The acetonitrile volume of was varied in range (400mL–700mL) at constant column temperature 40 °C, mobile phase pH 7 and 0.8mL/min flow rate. The volume 550mL was selected carefully as the best volume for optimal separation and better peak shape of (FIN). Column temperature was varied in range (25–40 °C) at 0.8mL/min flow rate, mobile phase pH 7 and 550mL acetonitrile. The investigation shows that high temperature (40 °C) provides more better peak shape and optimum retention time. So, 40 °C was selected as the optimum temperature. The flow rate was examined in range (0.8–1.2) mL/min at fixed column temperature 40 °C, pH 7 and 550mL acetonitrile. It was observed that flow rate below (1mL/min) provided sharp peak with reasonable retention time of (FIN) peak. So, 0.8mL/min was selected efficiently as the optimum flow rate. The system suitability parameters for estimation of (FIN) using the developed method were shown in **Table **S1.

### Method validation

The proposed technique was validated for the assay of (FIN) according to ICH guidelines^24^ and for testing of unspecified impurities according to the ICH Q3(B)guidelines^17^.

Validation of related substances determination is the more complex than other validation types used for quality control or assay of active pharmaceutical ingredients^25^. However, verification of these methods used for determination of unspecified impurities must be performed according to the ICH Q3(B)guidelines^17^. The validation of unidentified impurities determination procedures should be clear, precise and accurate because the total amount of unidentified impurities is typically higher than the total amount of known or identified impurities^25,26^.

#### Linearity and range

As presented in **Table.1a** linear relationship established between the (FIN) peak area and different (FIN) concentrations in ranges (8–30 µg/mL) for assay. A linearity range (0.2–1.4) µg/mL of FIN, covering concentrations of identification limit and the reporting threshold, was established for related substances assay method. The regression parameters presented in Table 1 indicate good linearities where r more than 0.999.

#### Limits of detection and quantitation

The following Eqs. (1) and (2) were used to calculate the LOD and LOQ of (FIN) for assay and for determination of unspecified impurities in accordance with the ICH guidelines^17,24^ as shown in Table 1. LOQ is necessary also to evaluate the precision and trueness to validate it.

1 $$\:LOD = 3.3\times\: o^,/S$$

2 $$\:LOD = 10\times\: o^,/S$$

where S is the slope and ơ is the standard deviation of y-intercept of regression lines.

**Table 1:** Regression parameters for (FIN) using the proposed technique.

	Assay of (FIN)	Determination of unspecified impurities
Concentration range (µg/mL)	8–30	0.2–1.4
r	0.99960	0.9997
a	41932.771	2304.057
b	36321.463	40459.857
S_a_	28919.871	771.619
S_b_	1345.609	862.696
S_(y/x)_	27356.418	912.992
LOD (µg/mL)	2.628	0.063
LOQ (µg/mL)	7.962	0.191

a, intercept; b, slope; r, correlation coefficient. S_a_, standard deviation of intercept; S_b_, standard deviation of slope; S_y/x_, residual standard deviation. LOD, limit of detection; LOQ, limit of quantitation.

#### Trueness

Trueness of the developed procedures was estimated by calculation of mean %recoveries of (FIN) using three determinations of different three concentrations within the linearity ranges (8, 12 and 20 µg/mL) for assay and (0.2, 1, 1.2 µg/mL) for determination of unspecified impurities. **Table S2** presented the calculated values of mean %recovery of (FIN), all results were found to be within compendial tolerance (98–102%).

#### Precision

Precision was assessed for both (FIN) assay and unspecified impurities determination by calculating (S.D) and (%RSD) values for three determinations of three different concentration levels of (FIN) on the same day and on three consecutive days for intra and inter-day precision, respectively. As presented in **Table S3**, all %RSD values were less than 2 confirming the good intra and inter day precisions of the proposed method.

#### Selectivity

Selectivity for assay procedures is confirmed regarding to ICH guidelines^24^by the comparison of the chromatograms obtained with the pure standard solution of (FIN), Nexifinerenone^®^ tablet 10 mg/tablet solution, inactive ingredients or placebo solution and diluent.

The chromatograms of dispersed inactive ingredients (placebo solution) and diluent solutions show no peaks at the previously mentioned retention time of (FIN).

This can be considered as a proof that the analytical method is unaffected by the different excipients present in dosage forms or the inactive ingredients do not interfere with the determination of (FIN) in Nexifinerenone table. The excipients do not interfere with the (FIN) peak as shown in **Figure S2**.

This is the same case when comparing the chromatograms of test or tablet solution of 0.5 mg/mL FIN, reporting threshold solution 0.0005 mg/mL FIN, identification or specification solution 0.001 mg/mL FIN, placebo and diluent solutions. The chromatograms of inactive ingredients (placebo solution) and diluent solutions show no interference or no peak at the previously mentioned retention time of (FIN) as presented in **(Figure S3 a-e)**.

#### Robustness

The robustness assists the ability of method to persist unaffected by small, but deliberate parameters change and indicates the reliability of method. The proposed method was robust as it was unaffected by the small deliberate parameter’s variations. The S.D. and %RSD values of %recoveries were less than 2 which indicate the robustness of method as presented in **Table S4**.

### Determination of unspecified impurities in FIN tablet

The diluent, placebo, solution 1 (sample or test solution), solution 2 (identification or specification limit 0.2%) and solution 3 (reporting threshold 0.1%) were injected under the specified chromatographic conditions. Then after examination of the chromatogram of solution 1 (test solution), any peak at the same retention time in placebo or blank solution was discarded. Also, any peak with area below the peak area of reporting threshold solution was discarded. the impurities that are detected in level exceed the reporting thresholds are the only impurities that can be considered as actual impurities according to ICHQ3(B) guidelines^17^. The identification is essential only when the levels of these impurities are expected to be above the specification or identification thresholds in the final drug product^17^. The percentage of each impurity in the portion of tablets taken was calculate the by the following formula:

3 $$\%\:{\rm impurity\:in\:the\:portion\:of\:tablets\:=\:(Pi\:/\:Ps)\:*(Cs\:/\:Ct)\:*100}$$

P_i_ = peak response of any unspecified peak from solution (1).

P_s_ = peak response of (FIN) from solution (2).

C_s_ = concentration of (FIN) in solution (2) (mg/mL).

C_t_ = concentration of (FIN) in solution (1) sample solution.

After examination of test solution (solution 1) of Nexifinerenone^®^ tablet Fig. 3, **(c)**, there is no any peaks of the unspecified impurities that are exceeded reporting or identification thresholds.

### Results of forced degradation studies

**Fig. 2:**
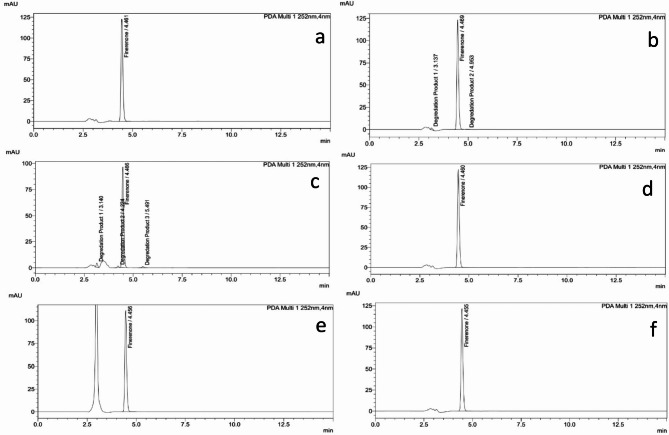
chromatograms of** a**: unstressed 20 µg/mL (FIN) solution,** b**: acidic degradation conditions (0.1 N HCl),** c**: alkaline degradation conditions (0.1 N NaOH),** d**: photo degradation for 2 h,** e**: oxidative degradation,** f**: heat degradation.

**Fig. 3:**
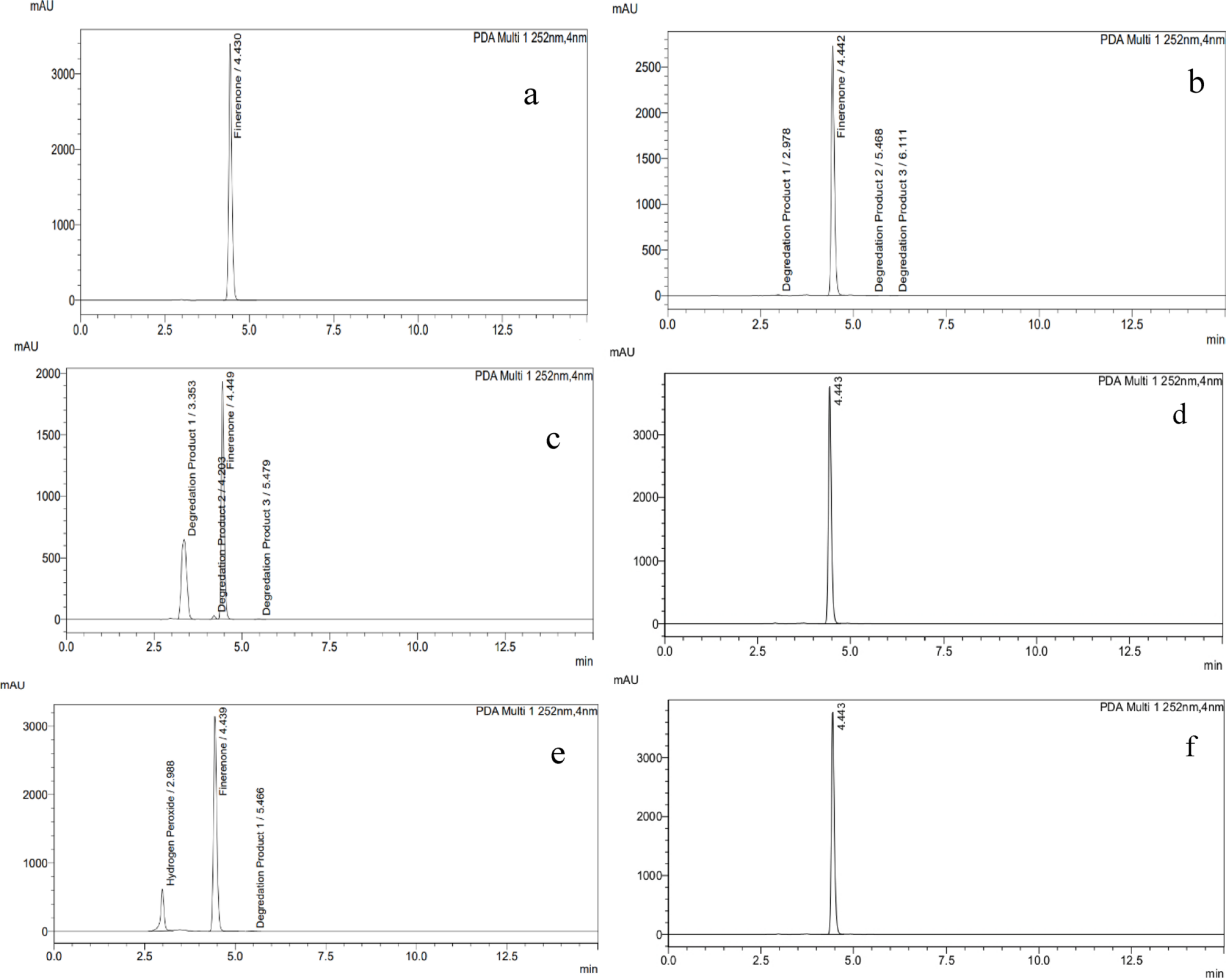
chromatograms of** a**: unstressed 0.5 mg/mL (FIN) solution of,** b**: acidic degradation,** c**: alkaline degradation,** d**: photo-degradation,** e**: oxidative degradation and** f**: heat degradation.

Specificity is confirmed as per ICH guidelines^17,24^ by the comparison of chromatograms of a freshly prepared non-stressed standard solution of 20 µg/mL (FIN) for assay and 0.5 mg/mL (FIN) for unspecified impurities determination as show in Figs. 2 and 3**(a)** with solutions exposed to degradation (alkaline hydrolysis, acid hydrolysis, oxidative, photo and heat degradation).

#### Acid hydrolysis

As presented in Fig. 2**(b)** and Table 2, there was a very slight acidic degradation of (FIN) as the (FIN) %recovery was reduced about 7.5% with presence of two new degradation peaks. For unspecified impurities determination, the chromatogram of acidic degradation (Fig. 3, b**)** showed about 24% reduction and presence of and 3 small degradation peaks as shown in Table 3.

#### Alkaline hydrolysis

The (FIN) chromatogram shows significant degradation and high sensitivity of (FIN) to alkaline degradation. This was indicated by decrease of (FIN) %recovery about 28.35% and 3 small peaks were appeared as shown in Fig. 2**(c) and** Table 2. For unspecified impurities determination, the chromatogram of alkaline degradation shows the maximum %degradation about (49%) and appearance of 3 small peaks as shown in Table 3**and** Fig. 3**(c)**.

#### Photo degradation

The (FIN) chromatograms under photo degradation conditions were showed good stability as shown in **(**Figs. 2 and 3 and d**)**. This was confirmed by the (FIN) peak area which doesn’t change and no any new degradation peak appeared after being exposed to the day light for 24 h as shown in and Tables 2 and 3.

#### Oxidative degradation

The (FIN) chromatogram under oxidative degradation conditions was showed about 10% decrease of peak area and no appearance of new degradation peaks as presented in Fig. 2**(e)** and Table 2. For unspecified impurities determination, the (FIN) chromatogram under oxidative degradation was showed slight degradation about 6% and presence of only one small as presented in Table 3**and** Fig. 3**(e)**.

#### Heat hydrolysis

As shown in Figs. 2 and 3**(f)**, and Tables 2 and 3, the (FIN) chromatograms under heat degradation conditions were showed good stability because their peak area doesn’t decrease and no presence of any new degradation peak.

**Table 2:** Forced degradation study results for (FIN) determination.

Degradation conditions	Unstressed	Acidic	Alkaline	Oxidation	Heat	Photolytic
Conditions	--	(0.1 N HCl, 2 h, 80 °C)	(0.1 N NaOH, 2 h, 80 °C)	(0.5mL 30%H_2_O_2_, 30 min.)	(80 °C, 2 h.)	(Day light, 24 h.)
Peak Area	822,801	761,184	589,567	740,491	815,608	816,833
% Found	100.00%	92.511	71.654	89.996	99.126	99.275
% Degradation	--	7.489	28.346	10.004	0.874	0.725
New peaks	--	2 Peaks	3 Peaks	--	--	--

**Table 3:** Forced degradation studies results of (FIN) related substances determination.

Degradation conditions	Unstressed	Acidic	Alkaline	Oxidation	Photolytic	Heat
		(0.1 *N* HCl, 2 h, 80 °C)	(0.1 *N* NaOH, 2 h, 80 °C)	(0.5mL 30%H_2_O_2_, 30 min.)	(Day light, 24 h.)	(80 °C, 2 h.)
Peak Area	20,985,072	15,948,748	10,645,791	19,701,028	20,816,602	20,810,389
% Found	100.00%	76.000	50.730	93.881	99.197	99.168
% Degradation	--	24.000	49.270	6.119	0.803	0.832
Number of new peaks	--	3 Peaks	3 Peaks	1 Peak	--	--

### Assay of nexifinerenone^®^ film coated tablets

**Fig. 4:**
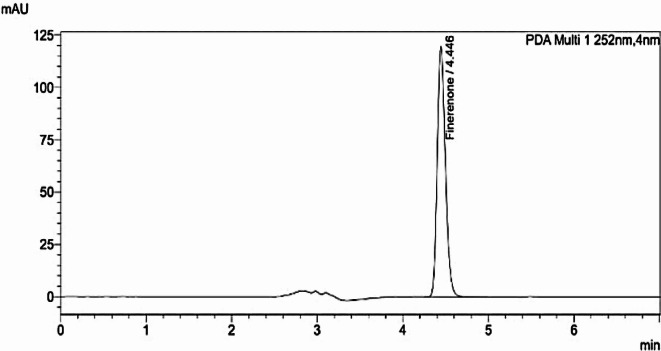
Chromatogram of (FIN) in Nexifinerenone^®^ tablets (10 mg/tablets).

The developed technique was successfully applied for the estimation of (FIN) in Nexifinerenone^®^ tablets available in Egyptian markets and gave reasonable results (Fig. 4).

As presented in Table 4, The results of proposed approach were compared to the results of published approaches^9^ for accuracy and precision based on T-test and F-test at 95% confidence level, respectively. There was no significant difference between the developed and published approach^9^ as the calculated results did not exceed the tabulated results.

**Table 4:** Assay results of (FIN) in Nexifinerenone^®^ tablets using proposed and reported^9^ methods.

	Proposed method	Reported method^9^
Mean (Ẋ)	100.742	100.102
S. D	0.890	0.471
%RSD	0.883	0.470
T_cal_	1.559	T _tab_: 2.306
F_cal_	3.571	F _tab_: 5.0503

S.D: standard deviation, RSD: relative standard deviation, t_cal_: calculated t-value,

t_tab_: tabulated t-value, F_cal_: calculated F-value, F_tab_: tabulated F-value.

### Green profile of the procedure

**Fig. 5:**
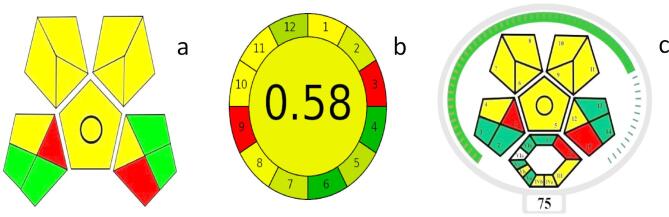
Greenness assessment results using Complex GAPI (**a**), AGREE (**b**) and Complex MoGAPI (**c**) methods.

Complex (GAPI)^20^Complex MoGAPI^21^ and Analytical GREEnness (AGREE)^22^ methodologies were used to evaluate the suggested method’s greenness^23^. Complex GAPI gives a pictogram evaluation of greenness across several analytical stage based on sample preparation steps, reagents used, instrumentation, waste treatment, and energy consumption. The resulting pictogram visually represents the method’s greenness profile with an emphasis on reagent toxicity and energy requirements^27^ (Fig. 5, a). The software for the Complex GAPI is available under the open-source MIT license and can be downloaded from https://mostwiedzy.pl/complexgapi20. The AGREE approach provides a thorough framework for assessing sustainability from a variety of angles, including as economic and safety considerations^22^. The 12 principles of green analytical chemistry were assessed using the AGREE software^22^generating a circular pictogram that highlights areas of greenness and areas for potential improvement (Fig. 5, b). Freely available software for the AGREE makes the assessment procedure straightforward. It is open-source and downloadable from https://mostwiedzy.pl/AGREE^22^. Greenness evaluation using Complex GAPI; metric does not provide a total score to determine whether the method is truly green. So, a modified version, Complex MoGAPI^21^addresses this limitation was used for greenness assessment^21^ (Fig. 5, c). The software for the MoGAPI tool is also freely available (open source) at bit.ly/MoGAPI to facilitate application and method comparison^21^.

Results from all three evaluation tools were presented in **(**Fig. 5**)** consistently showed that our HPLC approach is substantially green, according to comparative analysis.

Green methods are not necessarily practical, so the practicality of the developed method was evaluated using BAGI^28^ (**Figure S4**,** a**) and CACI^29^ (**Figure S4**,** b**) methods.

**Table S5** presents a comparison between the proposed method and four reported stability indicating methods^8,10,12,13^. The comparison confirms the superiority and advantages of proposed method.

## Conclusion

A rapid and sensitive stability indicating RP-HPLC method was developed and validated for the analysis of (FIN) in their bulk and Nexifinerenone^®^ film coated tablets. The proposed approach was applied and validated for determination of (FIN) related substances and testing the presence of unspecified impurities in tablet dosage form. The comparison between the assay results found by the proposed approach to those found by the reported one showed good agreements. The stability studies of (FIN) were performed to approve the specificity of the developed method. Complex GAPI, Complex MoGAPI and AGREE methods were applied for greenness assessment of the proposed method.

## Electronic supplementary material

Below is the link to the electronic supplementary material.

Supplementary Material 1

## Data Availability

Data is provided within the manuscript or supplementary information files.
