# Supplementary material for: Stability indicating RP-HPLC method for estimation of finerenone and its related substances in new dosage form
Source: Sci Rep. 2025 Jun 20;15:20229. doi: 10.1038/s41598-025-07166-4 (PMC12181283; doi:10.1038/s41598-025-07166-4)
Supplement: Supplementary file 1 — Supplementary Material 1 [file 41598_2025_7166_MOESM1_ESM.docx]

**Supplementary figures**

**Figure captions**

1. **Figure S1:** Chemical structures of Finerenone (FIN).
2. **Figure S2**: Chromatograms of a; inactive ingredients (placebo) and b; 20µg/mL (FIN) tablet solution.
3. **Figure S3**: Chromatograms of a: reporting threshold solution (0.0005mg/mL), b: identification (specification) threshold solution (0.001mg/mL), c: test solution (0.5mg/mL), d: placebo solution and e: diluent solution (all under the stated separation conditions).
4. **Figure S4**: The applicability and practicality assessment of the developed method using BAGI (a) and CACI (b).

**Figure S1:** Chemical structures of Finerenone (FIN).


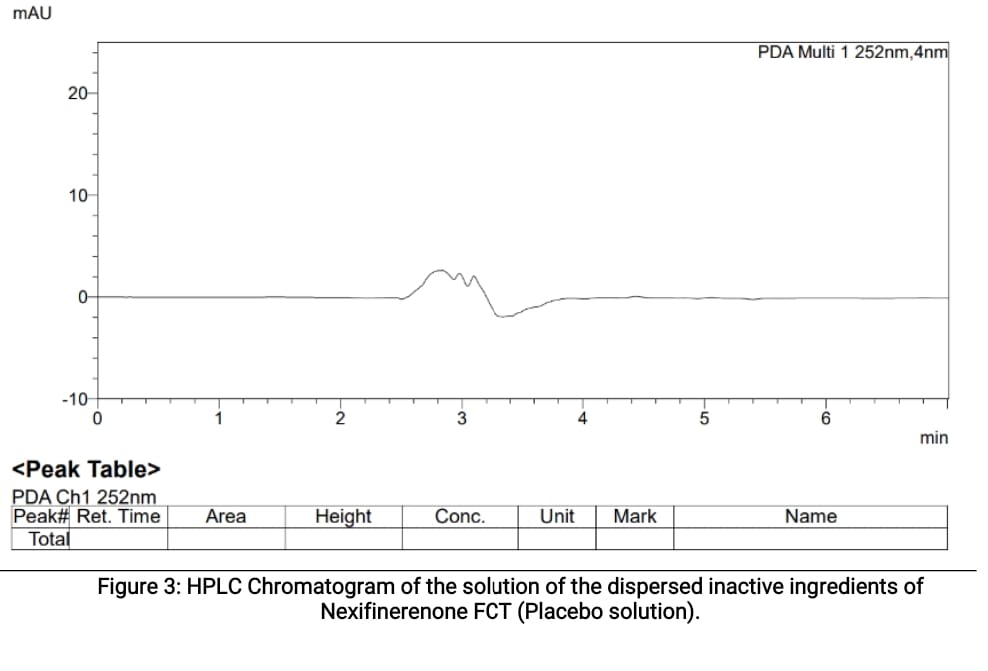


a

**
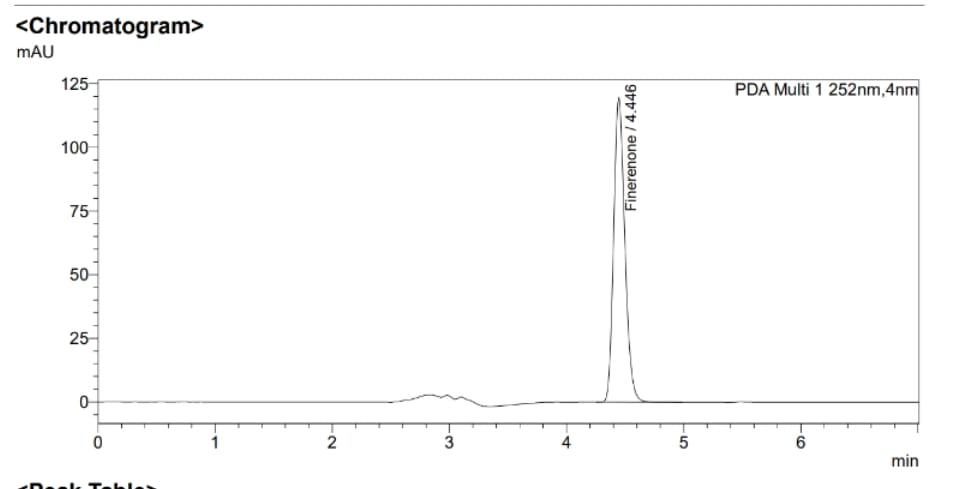
**

b

**Figure S2**: Chromatograms of a; inactive ingredients (placebo) and b; 20µg/mL (FIN) tablet solution.


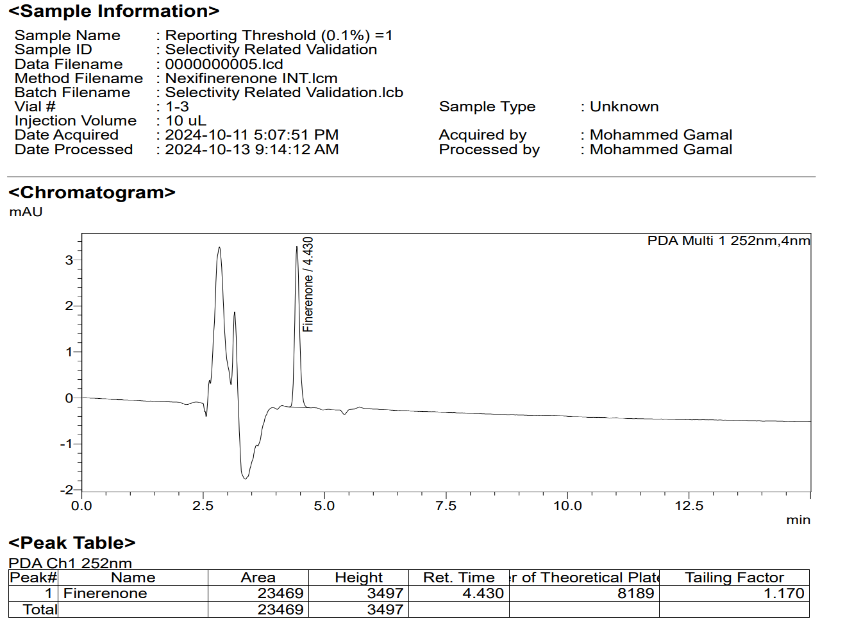


a


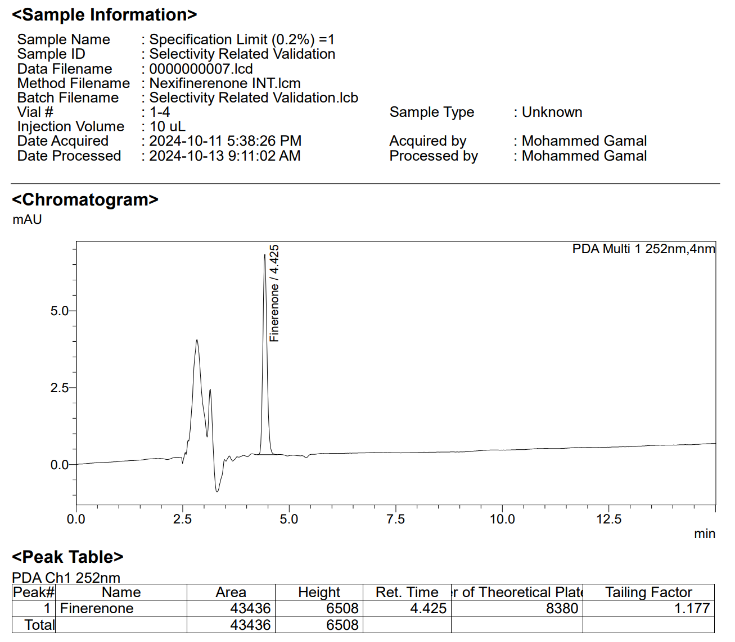


b


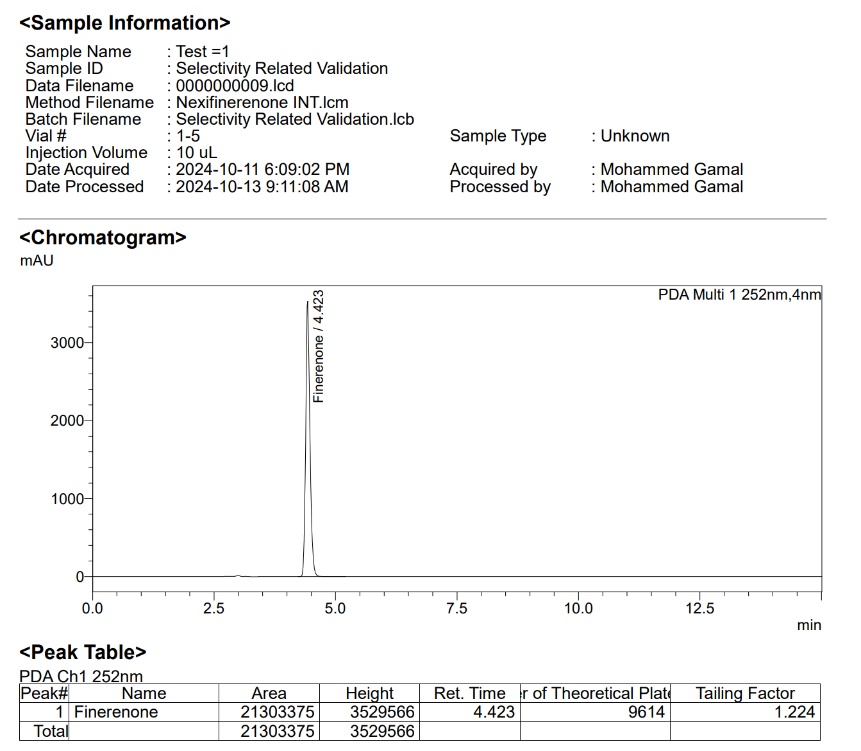


c


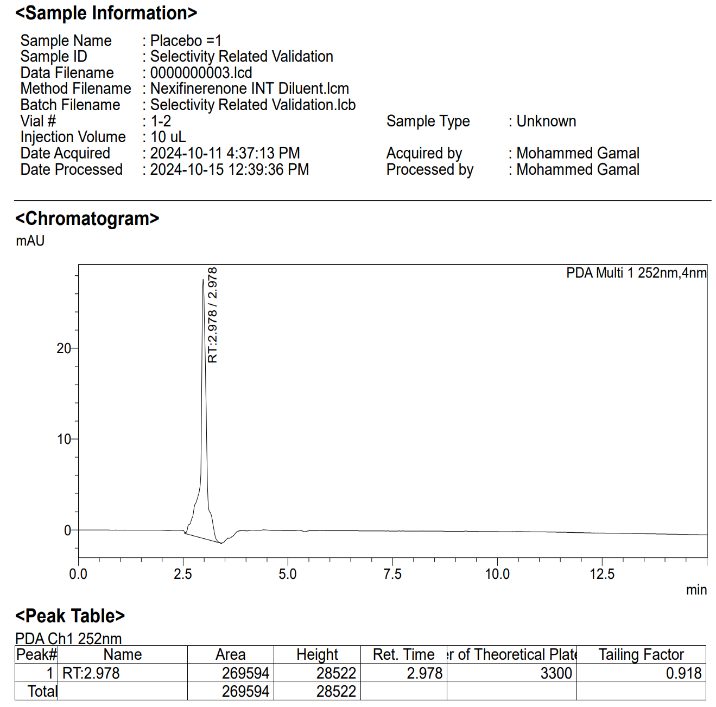


d


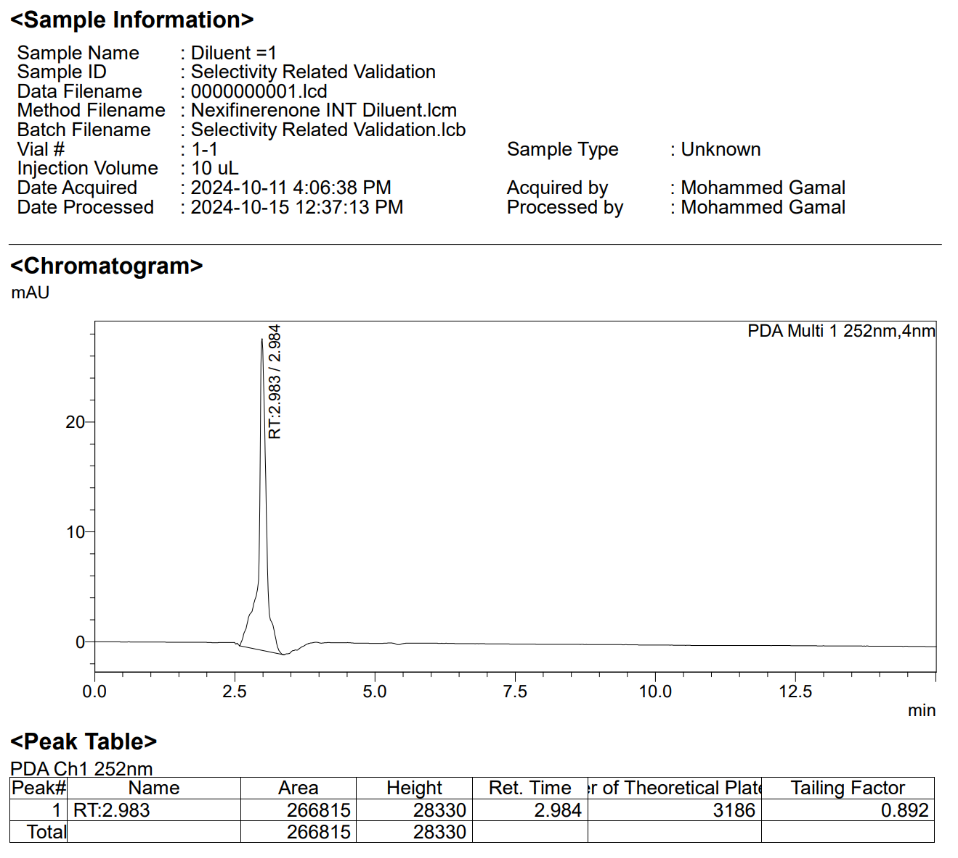


e

**Figure S3**: Chromatograms of a: reporting threshold solution (0.0005mg/mL), b: identification (specification) threshold solution (0.001mg/mL), c: test solution (0.5mg/mL), d: placebo solution and e: diluent solution (all under the stated separation conditions).


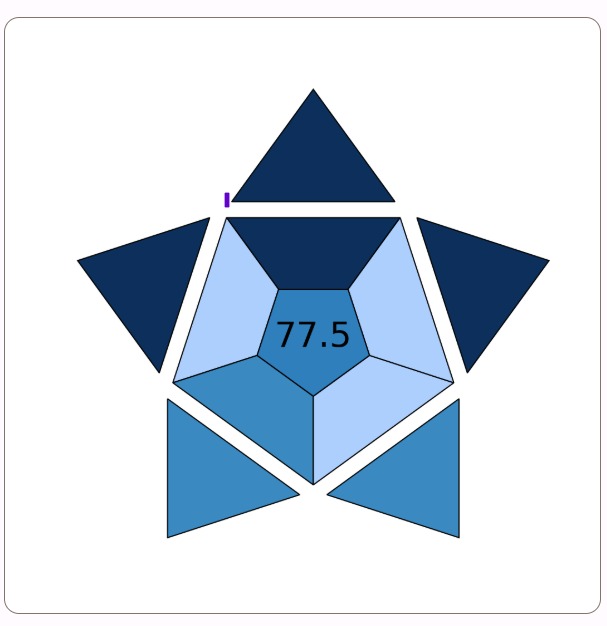


a


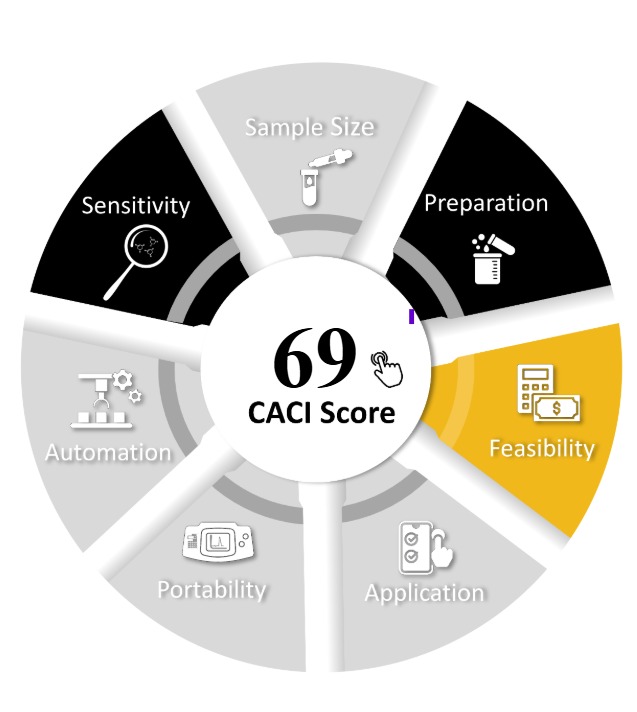


b

**Figure S4**: The applicability and practicality assessment of the developed method using BAGI (a) and CACI (b).

**Supplementary Tables**

1. **Table S1:** System suitability parameters for estimation (FIN).
2. **Table S2**: Accuracy results for (FIN) determination using proposed method.
3. **Table S3**: Results of precision of (FIN) using proposed method.
4. **Table S4:** Results of method robustness for estimation of (FIN).
5. **Table S5**: Comparison between the proposed stability indicating method and four reported stability indicating methods.

**Table S1:** System suitability parameters for estimation (FIN).

| Parameters | Results | Reference values |
| --- | --- | --- |
| Retention time t_R_ (min.) | 4.433 | -- |
| Tailing factor | 1.173 | <2 |
| Number of theoretical plates | 8453 | more is better |

**Table S2**: Accuracy results for (FIN) determination using proposed method.

| Assay of (FIN) | | | Determination of unspecified impurities | | |  |
| --- | --- | --- | --- | --- | --- | --- |
| Taken Conc.  (µg/ mL) | Recovered Conc. (µg/ mL) | Recovery % | Taken Conc. (µg/ mL) | Recovered Conc. (µg/ mL) | Recovery % |  |
|  |  |  |  |  |  |  |
| 8 | 7.996 | 99.950 | 0.2 | 0.201 | 100.500 |  |
| 12 | 11.837 | 98.642 | 1 | 0.985 | 98.500 |  |
| 20 | 20.126 | 100.630 | 1.2 | 1.18 | 98.333 |  |
| Mean % | 99.741 | | Mean % | 99.111 | |  |
| S.D. | 1.011 | | S.D. | 1.206 | |  |
| RSD % | 1.013 | | RSD % | 1.217 | |  |

S.D: standard deviation, RSD: relative standard deviation.

**Table S3**: Results of precision of (FIN) using proposed method.

| Assay of (FIN) | | | | | Determination of unspecified impurities | | | | |
| --- | --- | --- | --- | --- | --- | --- | --- | --- | --- |
| Taken conc.  (µg/ mL) | Intra-day precision | | Inter-day precision | | Taken conc.  (µg/ mL) | Intra-day precision | | Inter-day precision | |
|  | Mean conc. found | %RSD | Mean conc. found | %RSD |  | Mean conc. found | %RSD | Mean conc. found | %RSD |
| 8 | 8.112 | 0.789 | 7.992 | 0.824 | 0.2 | 0.204 | 0.108 | 0.211 | 0.502 |
| 12 | 12.065 | 0.522 | 12.012 | 0.753 | 1 | 0.983 | 0.201 | 0.995 | 0.422 |
| 20 | 20.250 | 0.235 | 20.121 | 0.523 | 1.2 | 1.180 | 0.031 | 1.186 | 0.214 |

S.D: standard deviation, RSD: relative standard deviation

**Table S4:** Results of method robustness for estimation of (FIN).

| Parameters | Conditions | Mean % recovery | S.D. | %RSD |
| --- | --- | --- | --- | --- |
| Flow rate | 0.7 mL/min | 100.152% | 0.218 | 0.218 |
|  | *0.8 mL/min |  |  |  |
|  | 0.9 mL/min |  |  |  |
| Temp. | 38 °C | 100.379% | 0.123 | 0.123 |
|  | *40 °C |  |  |  |
|  | 42 °C |  |  |  |
| pH | 6.9 | 101.132% | 0.321 | 0.317 |
|  | * 7 |  |  |  |
|  | 7.1 |  |  |  |

*Indicate the optimum chromatographic conditions, S.D: standard deviation, RSD: relative standard deviation

**Table S5**: Comparison between the proposed stability indicating method and four reported stability indicating methods.

|  | Proposed method | Reported method (8) | Reported method (10) | Reported method (12) | Reported method (13) |
| --- | --- | --- | --- | --- | --- |
| Purpose | a stability indicating RP-HPLC approach for quantitation of (FIN) in bulk and new tablet dosage form in addition, applying this approach for the determination of unspecified impurities in drug tablets Nexifinerenone® 10mg/tablet. | Measure finerenone (FIN) &  analysis of degraded samples | estimation of FIN in bulk form using analytical quality by design approach.  and Test drug's stability under various stress situations | Estimation of FIN in bulk form using analytical quality by design approach.  Test drug's stability under various stress situations | stability indicating (RP-HPLC) for the estimation of FIN |
| Linearity ranges | (8-30µg/mL) for assay of (FIN) and (0.2-1.4µg/mL) for (FIN) unspecified impurities determination. | 80–120μg/mL | -- | 1–50 µg/mL | 5–45  µg/mL |
| LOD and LOQ | LOQ were 8.093 for assay and 0.206 for unspecified impurities determination.  LOD were 2.671 for assay and 0.068 for unspecified impurities determination. | LOQ at 1.634 and LOD at 0.539 | LOD and LOQ values were 0.261 µg/ml and 0.818µg/ml | LOD and LOQ for the drug Finerenone was found to be 0.02 µg/mL and 0.04 µg/mL | LOQ 3.112 µg/mL and LOD 1.022 µg/mL |
| Greenness assessment tools | GAPI, AGREE and complex MoGAPI | -- | -- | -- | -- |
| Method applicability tools | BAGI and CACI | -- | -- | -- | -- |
| Mobile phase | mixture of 450mL water, 550mL acetonitrile and 10 mL triethylamine with pH adjusted to 7. | Acetonitrile: water (50:50 v/v) | KH_2_PO_4_ buffer and methanol (50:50%V/V) | Acetonitrile and Ortho-Phosphoric acid (70:30% v/v). | 0.1% Trifluoro acetic Acid: Acetonitrile (70:30) |
